# A Cone-Beam Computed Tomography Scanning of the Root Canal System of Permanent Teeth among the Moscow Population

**DOI:** 10.1155/2018/2615746

**Published:** 2018-09-25

**Authors:** Svetlana Razumova, Anzhela Brago, Lamara Khaskhanova, Ammar Howijieh, Haydar Barakat, Ashot Manvelyan

**Affiliations:** Peoples' Friendship University of Russia (RUDN University), 6 Miklukho-Maklaya Street, Moscow 117198, Russia

## Abstract

**Background:**

Successful endodontic treatment requires a significant knowledge of root canal anatomy. The aim of this study was to evaluate the root and root canal number of permanent teeth among the Moscow population using cone-beam computed tomography (CBCT) scanning.

**Materials and methods:**

300 CBCT images of subjects were analyzed to study the anatomy of roots and root canal system of each tooth. The collected data were analyzed using IBM SPSS statistics software 22.0 version.

**Results:**

The maxillary incisors and canines had one root with one canal in 100%. Maxillary premolars had one root with one or two canals and two roots with two canals, while mandibular premolars were single-rooted with one or two canals. Maxillary first and second molar had three separated roots, and the prevalence of four canals was more often in first molars. Mandibular molars had two roots with different number of canals.

**Conclusion:**

The root canal system varies greatly among populations and even in different individuals within the same population; thus, using CBCT scanning is an effective technique in investigating the root canal system.

## 1. Background

The success of endodontic treatment depends on the precise knowledge of root and root canal anatomy, which is an important challenge due to the complexity of the root canal system and the anatomical variations [1, 2]. This knowledge helps the clinicians in endodontic treatment planning and decreases the percent of endodontic treatment failure. Many studies have been conducted to determine the anatomy and morphology of root canal system using different techniques [3–9]. Recently, with the development of cone-beam computed tomography (CBCT) technique, it became possible to study the anatomy of teeth, due to the high-quality three-dimensional (3D) images obtained from CBCT. This image allows studying the anatomy of the jaws, the morphology of teeth, and their root canal system [3]. Many studies have used this technique to find out the anatomy of root canal system especially in the molar regions [3, 6, 8].

Root canal morphology has been classified using different ways by several investigators in the literature [10–12]. Weine et al. [12] classified it into four types depending on the pattern of division of the main root canal of a tooth along its course from the floor of the pulp chamber to the root apex. Vertucci [10] also classified the root canal morphology in a more descriptive manner into eight types. This classification has been widely used by many researchers to classify the canal system of different teeth.

It is known that the number of teeth roots and the anatomy of root canal system vary among the world population, and to date, there are still no studies about the anatomy of roots and root canals in Russian Federation, where dental practitioners still use the data obtained from foreign authors on the anatomy of root canals in planning the endodontic treatment. The aim of this study was to investigate the anatomy of root and root canal system among the Moscow population using CBCT technique.

## 2. Materials and Methods

300 subjects aged 20–70 years were enrolled in this study, from those attending the diagnostic center (LLC Zolotoye Secheniye) for 3D radiological scanning in the period between January 2017 and November 2017. The study protocol was approved by the Ethics Committee in People's Friendship University of Russia (RUDN University), Moscow, Russia. A written consent was signed by all subjects, including who participated in this study. CBCT images were taken using a 3D eXam® dental tomography scanner (KaVo, Biberach, Germany) with standard exposure settings (23 ∗ 17 cm field of view; 0.3 mm voxel size; 110 kv; 1.6–20 s) and were viewed by three examiners in a semidark room using I-CAT viewer software (version 10, Hatfield, England). All teeth were evaluated in axial, coronal, and sagittal planes, and the number of roots and the number of canals in each tooth were recorded. The presence of additional canals especially in the molar regions was recorded.

### 2.1. Statistical Analysis

Data were processed using the software (SPSS v 22 for win, IBM, Chicago, IL), and descriptive data were processed to analyze the percentage of roots and root canals in each tooth.

## 3. Results

300 CBCT scans of 300 subjects with mean age of 49.91 ± 14.01 were analyzed. All the maxillary incisors had one root with 100% and one canal with 100%. First and second maxillary molars had three roots with 100%, and third molar recorded one root in 33.7% and three roots in 47.9%. Data for maxillary teeth including the number of roots and root canals are shown in (Table 1).

For mandibular teeth, data were the following: central and lateral incisors and canines showed one root and one canal in 99.4%, 99.2%, and 99.8%, respectively. First molars had two roots in 100%, and second molars had one root in 0.5% and two roots in 99.5%. Data for mandibular teeth are shown in (Table 2).

## 4. Discussion

The knowledge of the anatomy of the root canal system is the gold standard in successful endodontic treatment, and studying root and canal morphology has endodontic and anthropological significance. This study evaluated the roots and root canal system among the Moscow population using CBCT scanning. The advantages of CBCT scanning are its high accuracy, minimal distortion, and 3D visualization. Moreover, it is a nondestructive technique and can provide specific data [3].

When analyzing the anatomy of the maxillary anterior teeth, 510 central incisors, 500 lateral incisors, and 540 canines were recorded, and one root with one canal was identified in 100% of cases. These data are comparable with the results obtained from other studies on the anatomy of central incisors in USA [10], Mexico [11], Turkey [13], and Chennai urban [14]. The presence of two canals in lateral incisors was found in 13.7% of cases in a study by Calişkan et al. (Turkey) [13], in 9.5% of cases by Sert and BayirliIn (Turkey) [15], and in 2% in a study by Jain et al. (Chennai urban) [14]. The prevalence of two canals in canines was 3.9%, 2–4%, 4%, and 18.4% in studies by Calişkan et al. [13], Sert and Bayirli [15], Jain et al. [14], and Amardeep et al. [16] respectively. This difference in results could be attributed to the evaluation method, sample size, and racial differences.

According to the current study, the anatomy of root canals of maxillary first premolar was variable, two roots were identified in 91.3% of cases and 8.7% of cases were one rooted, and the first premolar recorded one root and two canals in 2.6% and one canal in 6.1% of cases. Similar data obtained in the studies of Bulut et al. (Turkey) [17], Pecora et.al. (Brazil) [18], Kerekes and Tronstad (Norway) [19], Green [20] (USA), Ok et al. (Turkey) [21], and Burklein et.al. (Germany) [22]. Pineda and Kuttler (Mexico) [11] have revealed in their studies a single-canal first premolar in 50.1% and two canals in 49.4% of cases.

The maxillary second premolar with one root was determined in 26.5% of observations and with two separated roots in 73.5% of cases according to the data of the current study, and two canals were recorded in 73.5% of cases. This coincides with the results of Awawdeh et al. (Jordan) [23], where one canal was found in 13.8% and 84.1% had two canals; the results of Elnour et al. (Saudi Arabia) [24], where two canals were recorded in 65% of cases; and the results of Bürklein et al. (Germany) [22], where 56.3% of cases had two canals. Bulut et al. (Turkey) [17] recorded one canal in 82.1% of the cases and two canals in 17.8%.

The maxillary first molar was analyzed by all researchers in detail by the number of canals in each root. Three separated roots were identified in 100% of cases. The number of canals varied from 3 to 5. The four-canal root system occurred in 59.8% of cases, and the localization of the two canals takes place more often in the mesiobuccal root (MB) and also occurred in the distobuccal root (DB). These results are comparable with the results of Pomeranz and Fishelberg (USA) [25], where 48% of cases had two canals in MB. Wasti et al. (Pakistan) [26] recorded a percent of two canals in MB in 43.4%, and Pineda and Kuttler (Mexico) [11] defined two canals in 48.5% of cases. This significantly varies from the results of Imura et al. (Japan) [27], where 88.2% of cases had two canals in the MB, and of Kim et al. (Korea) [8], where two canals in MB were found in 63.59% and 1.25% in DB. Ghoncheh et al. (Iran) [28], Martins et al. (Caucasian population) [29], Ratanajirasut et al. (Thai) [30], Silva et al. (Brazil) [31], and Guo et al. (North America) [32] recorded four-canal system in 46%, 71%, 63.6%, 42.63%, and 68.2% of cases, respectively. Ceperuelo et al. (Spain) [33] defined four-canal system in 92.3% of cases.

The maxillary second molar consisted of three separated roots in 100% of cases, and the prevalence of four canal systems was identified in 51.5% of cases (two canals in MB). This agreed with the published studies of Shalabi et al. (Ireland) 50% [34], Kulild and Peters (USA) 45.8% [35], Alavi et al. (Thailand) 44.6% [36], Kim et al. (Korea) 34.39% [8], Martins et al. (Caucasian population) 44% [29], and Ratanajirasut et al. (Thai) 29.4% [30] of the prevalence of two canals in MB. Ghoncheh et al. (Iran) [28], Silva et al. (Brazil) [31], Ceperuelo et al. (Spain) [33], Betancourt et al. (Chili) [37], Al-Fouzan et al. (Saudi Arabia) [38], and Li et al. (China) [39] reported 14%, 32%, 75%, 48%, 19.7%, and 41.3%, respectively, of the prevalence of MB2 in maxillary second molar.

The maxillary third molar in our study was defined as single-rooted in 47.9% and three-rooted in 52.1%, and the system of root canals could consist of one, two, three, or more canals as shown in (Table 1). Similar results were found in a study of Sidow et al. (USA) [40], in which, it was found 15% of third molars had one root and 45% had three roots, and the number of canals ranged from 1 to 6 in teeth with one root and 2 to 5 in teeth with 3 roots. A study by Tomaszewska et al. (Poland) [41] reported the one-rooted maxillary third molar in 38.5% of cases and 61.5% were three-rooted. The prevalence of one, three, and four canals were 23.1%, 46.1%, and 15.4%, respectively. Singh and Pawar (India) [42] found three-canal system of maxillary third molar in 43% of cases and four-canal system in 5%.

In this study, for mandibular teeth, it was identified in central and lateral incisors and canines, one canal in 99.4%, 99.2%, and 99.8%, respectively, and two canals in 0.6%, 0.8%, and 0.2% of cases, respectively. These findings are consistent with other studies, where one canal was recorded in central incisors in 99.7% in Madeira and Hetem (Brazil) [43] and 99% in Walker (China) [44]. For lateral incisors, studies have reported the presence of 2 canals in 1.3% (Mexico) [11], 0.8% (Brazil) [43], and 1% (China) [44]. The presence of 2 canals in canines was 20.48% and 20.4% in studies by Rahimi et al. (Iran) [45] and Amardeep et al. (India) [16].

The mandibular first premolar was found as single-rooted in 100% of cases. One canal was found in 89.2%, and two canals in 10.8% of cases. This coincides with the results of Bulut (Turkey) [17], in which 1 canal was recorded in 94.2%, Llena et al. (Spain) [46], Alhadainy (Egypt) [47], and Sobhani et al. (Iran) [48], and one canal was found in 78.1%, 61.2%, and 90.8%, respectively. The presence of 2 canals was recorded by Zillich and Dowson (USA) [49] in 18.9%, Abraham and Gopinath (Emirate) [50] 35 %, Dou et al. (china) [51] with 34.27%, Bürklein et al. (Germany) [22] with 21.9%, and Singh and Pawar (India) [52] with 22%.

The mandibular second premolar had one root in 99.8%, and it had two roots in one clinical case (0.2%). When analyzing the root canal system, two canals were defined in 9.7% of cases. Similar data were found in studies by Bulut et al. (Turkey) [17] and Llena et al. (Spain) [46] with 98.9% and 90.6%, respectively, for the prevalence of one canal. Al-Qudah and Awawdeh (Jordan) [53], Bürklein et al. (Germany) [22], Bolhari et al. (Iran) [54], and Singh and Pawar (India) [52] recorded the prevalence of 2 canals in 22.8%, 3.6%, 8.7%, and 42% of cases, respectively.

The mandibular first molar had two roots in 100% of the cases. And, the anatomy of the root canal system could be two-canal, three-canal, and four-canal. Most often, there are three canals in 78.6% of observations. Similar data were found in Vertucci (USA) 59% [10], Pineda and Kuttler (Mexico) 57% [11], Celikten (Turkey) 84.6% [55], of cases of three-canal first molars. Skidmore and Bjorndal (USA) [56] have revealed 55.5% of cases. Zhang et al. (China) [57] recorded 56% and Caputo et al. (Brazil) [58] recorded 75.1% of cases of three-canals in mandibular first molars. The four-canal system of the first molar was defined in our study in 20.9% of cases; it was also described in the literature by Wasti et al. (Pakistan) [26] in 43.3% of cases, Caputo et al. (Brazil) [58] in 23.7%, Hung et al. (Taiwan) [59] in 40.5%, Celikten et al. (Turkey) [55] in 10.4%, Al-Qudah and Awawdeh (Jordan) [60] in 45.8%, and Pattanshetti et al. (Kuwait) [61] in 46.4%.

When analyzing the structure of the root canal system of the mandibular second molar, it was defined to be a two-, three-, and four-canal systems. 82.2% of cases had three canals. The most similar data were found in Celikten et al. (Turkey) [55], where 85.9% of three canals were recorded, and Neelakantan et al. (India) [62], in which most of second molars had two roots with three canals in 87.8%. Zhang et al. (China) [57] and Pawar et al. (India) [63] defined 3-canal system in 46% and 35.5% of cases.

The mandibular third molar was revealed as two-rooted in most cases (80%), and the canal system could have two- and three-canals, and these results agreed with other studies of the anatomy of third molar, as in Kuzekanani et al. (Iran) [64] where 73% of cases had 2 roots, and a study by Sidow et al. (USA) [40], where 77% of two roots were defined.

## 5. Conclusions

The root canal system varies greatly among populations and regions and even in different individuals within the same population, and using CBCT scanning is an effective technique in investigating the root canal system.

## Figures and Tables

**Table 1:** Anatomy of roots and root canals in maxillary teeth.

Tooth	Number of studied teeth	Roots	*N* (%)	Canals	*N* (%)
Central incisor	510	1	510 (100%)	1	510 (100%)
Lateral incisor	500	1	500 (100%)	1	500 (100%)
Canine	540	1	540 (100%)	1	540 (100%)
First premolar	460	1	40 (8.7%)	1	28 (6.1%)
				2	12 (2.6%)
		2	420 (91.3%)	2	420 (91.3%)
Second premolar	423	1	112 (26.5%)	1	75 (17.7%)
				2	37 (8.8%)
		2	311 (73.5%)	2	311 (73.5%)
First molar	410	3	410 (100%)	3	163 (39.7%)
				4	245 (59.8%)
				5	2 (0.5%)
Second molar	435	3	435 (100%)	3	210 (48.3%)
				4	224 (51.5%)
				5	1 (0.2%)
Third molar	238	1	114 (47.9%)	1	33 (13.8%)
				2	28 (11.8%)
				3	53 (22.3%)
		3	124 (52.1%)	3	119 (50%)
				4	5 (2.1%)

**Table 2:** Anatomy of roots and root canals in mandibular teeth.

Tooth	Number of studied teeth	Roots	*N* (%)	Canals	*N* (%)
Central incisor	512	1	512 (100%)	1	509 (99.4%)
				2	3 (0.6%)
Lateral incisor	500	1	500 (100%)	1	496 (99.2%)
				2	4 (0.8%)
Canine	521	1	521 (100%)	1	520 (99.8%)
					1 (0.2%)
First premolar	490	1	490 (100%)	1	437 (89.2%)
				2	53 (10.8%)
Second premolar	443	1	442 (99.8%)	1	399 (90.1%)
				2	43 (9.7%)
		2	1 (0.2%)	2	1(0.2%)
First molar	407	2	407 (100%)	2	2 (0.5%)
				3	320 (78.6%)
				4	85 (20.9%)
Second molar	398	1	2 (0.5%)	2	2 (0.5%)
		2	396 (99.5%)	2	47 (11.8%)
				3	327 (82.2%)
				4	22 (5.5%)
Third molar	210	1	42 (20%)	1	1 (0.5 %)
				2	41 (19.5%)
		2	168 (80%)	2	45 (21.4%)
				3	123 (58.6%)

## Data Availability

The data used to support the findings of this study are included within the article.

## Abbreviations

CBCT: Cone-beam computed tomography
3D: Three-dimensional
MB: Mesiobuccal root
DB: Distobuccal root.
